# Predicting Postoperative Troponin in Patients Undergoing Elective Hip or Knee Arthroplasty: A Comparison of Five Cardiac Risk Prediction Tools

**DOI:** 10.1155/2022/8244047

**Published:** 2022-10-14

**Authors:** Merih T. Tesfazghi, Anne R. Bass, Noor Al-Hammadi, Scott C. Woller, Scott M. Stevens, Charles S. Eby, Mitchell G. Scott, Lindsey Snyder, Troy S. Wildes, Brian F. Gage

**Affiliations:** ^1^Department of Pathology and Immunology, Washington University in St Louis, St Louis, MO, USA; ^2^Department of Pathology, Rush University Medical Center, Chicago, IL, USA; ^3^Department of Medicine, Hospital for Special Surgery, Division of Rheumatology, Weill Cornell Medicine, New York, NY, USA; ^4^Department of Medicine, Washington University in St Louis, St Louis, MO, USA; ^5^Department of Medicine, Intermountain Medical Center, Salt Lake, UT, USA; ^6^Department of Medicine, University of Utah, Salt Lake, UT, USA; ^7^Department of Anesthesiology, Washington University in St Louis, St Louis, MO, USA

## Abstract

**Background:**

Elderly patients undergoing hip or knee arthroplasty are at a risk for myocardial injury after noncardiac surgery (MINS). We evaluated the ability of five common cardiac risk scores, alone or combined with baseline high-sensitivity cardiac troponin I (hs-cTnI), in predicting MINS and postoperative day 2 (POD2) hs-cTnI levels in patients undergoing elective total hip or knee arthroplasty.

**Methods:**

This study is ancillary to the Genetics-InFormatics Trial (GIFT) of Warfarin Therapy to Prevent Deep Venous Thrombosis, which enrolled patients 65 years and older undergoing elective total hip or knee arthroplasty. The five cardiac risk scores evaluated were the atherosclerotic cardiovascular disease calculator (ASCVD), the Framingham risk score (FRS), the American College of Surgeon's National Surgical Quality Improvement Program (ACS-NSQIP) calculator, the revised cardiac risk index (RCRI), and the reconstructed RCRI (R-RCRI).

**Results:**

None of the scores predicted MINS in women. Among men, the ASCVD (*C*-statistic of 0.66; *p*=0.04), ACS-NSQIP (*C*-statistic of 0.69; *p*=0.01), and RCRI (*C*-statistic of 0.64; *p*=0.04) predicted MINS. Among all patients, spearman correlations (*r*_s_) of the risk scores with the POD2 hs-cTnI levels were 0.24, 0.20, 0.11, 0.11, and 0.08 for the ASCVD, Framingham, ACS-NSQIP, RCRI, and R-RCRI scores, respectively, with *p* values of <0.001, <0.001, <0.001, 0.006, and 0.025. Baseline hs-cTnI predicted MINS (*C*-statistics: 0.63 in women and 0.72 in men) and postoperative hs-cTnI (*r*_s_ = 0.51, *p*=0.001).

**Conclusion:**

In elderly patients undergoing elective hip or knee arthroplasty, several of the scores modestly predicted MINS in men and correlated with POD2 hs-cTnI.

## 1. Introduction

Elderly patients undergoing elective total hip or knee arthroplasty are at risk for myocardial injury after noncardiac surgery (MINS) [1]. MINS, which is defined by cardiac troponin (cTn) values exceeding the 99th percentile threshold, presages cardiac morbidity and mortality [2–4]. For example, among patients undergoing major orthopedic surgery, those with MINS have a 10-fold increase in 30-day mortality [2]. However, most patients with MINS do not report symptoms of cardiac ischemia [1–6]. Furthermore, the risk of postoperative cardiac complications is proportional to the postoperative cardiac troponin (cTn) value [1, 3, 6–8] rather than to symptoms [1, 6]. Thus, accurate prediction of MINS and postoperative cTn levels could help assess the risk of elective arthroplasty and mitigate postoperative complications.

Several clinical scores are widely used to estimate cardiac complications in perioperative and non-perioperative settings. The revised cardiac risk index (RCRI) was developed to predict perioperative cardiac complications among patients undergoing noncardiac surgery [9]. However, it may not be an ideal predictor among patients undergoing elective joint arthroplasty because they have a low prevalence of most of the included risk factors: high-risk surgery, history of ischemic heart disease, heart failure, stroke, or significant renal insufficiency. The reconstructed RCRI (R-RCRI) is similar to the original RCRI except that it does not consider insulin therapy and uses glomerular filtration rate (GFR) < 30 mL/minute rather than creatinine >2 mg/dL [10]. The American College of Surgeon's National Surgical Quality Improvement Program (ACS-NSQIP) developed a calculator that uses 23 preoperative variables to quantify the risk of postoperative myocardial infarction. Whether the RCRI, R-RCRI, or ACS-NSQIP score is most accurate at predicting postoperative levels of cTn after joint arthroplasty is unknown [11–13]. The American College of Cardiology and American Heart Association 2014 guidelines recommend the use of either the RCRI or ACS-NSQIP to predict perioperative major cardiac complications [14].

Other scoring systems are used to predict long-term cardiovascular complications but have not been tested in the perioperative setting. The atherosclerotic cardiovascular disease (ASCVD) risk predictor was developed to estimate the 10-year risk of developing ASCVD events [15]. The Framingham risk score (FRS) was developed to predict the 10-year risk of developing cardiovascular disease [16]. Neither of these scores has been used to predict postoperative cTn levels.

In this study, we investigated the ability of the five widely known cardiac risk scores, alone or combined with baseline hs-cTnI levels, in predicting MINS and postoperative day 2 (POD2) hs-cTnI levels in elderly patients undergoing elective total hip or knee arthroplasty.

## 2. Methods and Materials

The study was reviewed and approved by the institutional review boards (IRBs) of the participating institutions. This study was ancillary to the Genetics-InFormatics Trial (GIFT) of Warfarin Therapy to Prevent Deep Venous Thrombosis study (NCT01006733). Written consent was obtained from all study participants. This study was performed in line with the principles of the Declaration of Helsinki.

### 2.1. Study Design

The design and outcome of the GIFT study were previously reported [17, 18]. Briefly, the GIFT study recruited patients (≥65 years of age) undergoing elective total knee or hip arthroplasty [17]. The trial evaluated the efficacy of genotype-guided warfarin dosing in reducing warfarin-related adverse events in an elderly arthroplasty population. All patients included in this ancillary study underwent arthroplasty and received at least one dose of warfarin.

### 2.2. Sample Collection and Storage

Baseline specimens were collected at three of the six GIFT study sites at a preoperative visit 5–14 days before surgery or on the day of surgery. Postoperative specimens were drawn on postoperative day 2 (POD2). All specimens were collected in citrate tubes and aliquot plasma specimens were frozen at −80°C until analysis. Immediately before analysis, frozen plasma specimens were thawed at room temperature (for 20–30 minutes), briefly vortexed, and centrifuged at 3000 RPM for 5–10 minutes. Paired plasma specimens were subjected to the same condition and analyzed together to avoid between-run variations.

### 2.3. Hs-cTnI and Lipid Assay

Of the 1650 GIFT trial participants, 808 patients had baseline and POD2 plasma specimens available and received perioperative warfarin. We measured baseline plasma concentrations of high-density lipoprotein cholesterol (HDL-C), triglyceride, and cholesterol on the Siemens Dimension® EXL 200 platform (Erlangen, Germany). We measured hs-cTnI concentrations on the Abbott Architect i2000_SR_ platform (Abbott Park, IL, United States). Per the manufacturer's package insert (research use only), the coefficient of variations (CV) of hs-cTnI at the 99th percentile thresholds of 16 ng/L for women and 34 ng/L for men was 5.3% and 3.5%, respectively. The limit of quantitation (LoQ) was 4.7 ng/L with a 10% CV. The limit of detection (LoD) for this assay ranged from 1.1 to 1.9 ng/L across reagent lots/instrument combinations with a 20% CV at 1.3 ng/L. In this study, troponin elevation was defined by the sex-specific 99th percentile thresholds (>16 ng/L for women; >34 ng/L for men) for the Abbott method as recommended [19].

### 2.4. Definition of Myocardial Injury after Noncardiac Surgery (MINS) and of Myocardial Infarction (MI)

MINS was defined as a rise in hs-cTnI from baseline (with or without ischemic symptoms) occurring on POD2 [3, 19] and exceeding the sex-specific cutoffs of 16 ng/L for women and 34 ng/L for men. No patients with MINS had a perioperative (within 2 days of surgery) pulmonary embolism or stroke. No participants with sepsis were recruited for this substudy, as sepsis can elevate levels of postoperative cTn [19]. Myocardial infarction was defined according to the 4th universal definition guideline, which is based on the presence of ischemic symptoms or abnormal ECG, and elevated troponin levels with at least one value above the 99th percentile [19].

### 2.5. Determination of Risk Scores

We calculated the risk scores as recommended. The ASCVD score used the following variables: age, diabetes, race, sex, smoking status, systolic blood pressure, treatment for hypertension, total cholesterol, and HDL cholesterol [15]. FRS used the following variables: age, diabetes, sex, smoking status, systolic blood pressure, and total and high-density lipoprotein cholesterol to quantify the risk of atherosclerotic cardiovascular disease, stratified by gender [16]. We calculated the American College of Surgery ACS-NSQIP score [15] by entering the required variables into their website, https://riskcalculator.facs.org/RiskCalculator/, without adding geriatric outcomes or surgeon-specific adjustments. Functional status (one of the variables used by ACS-NSQIP) was not collected. Therefore, patients who were prospectively identified as bedbound were classified as totally dependent; those who reported 2 or more falls in the past 12 months on the GIFT intake form were classified as partially dependent. The remaining patients were classified as independent. RCRI used the following variables: high-risk surgery, history of ischemic heart disease, history of heart failure, history of cerebrovascular disease, diabetes mellitus requiring treatment with insulin, and preoperative serum creatinine >2 mg/dL [9]. The elective joint arthroplasty was considered an intermediate-risk procedure. R-RCRI used the history of ischemic heart disease, history of heart failure, history of cerebrovascular disease, high-risk surgery, or preoperative GFR < 30 mL/minute [10]. GFR was calculated from the baseline creatinine using the modification of diet in renal disease (MDRD) equation [20].

### 2.6. Statistical Analysis

Baseline hs-cTnI was undetectable in one participant and unavailable in 24 patients due to insufficient specimens. For these patients, the missing values were imputed to be normal (1.0 ng/L). We compared baseline continuous variables using the Mann–Whitney *U* test and categorical variables using the chi-square (*x*^2^) or Fisher's exact test, as appropriate. We used the Cochran–Armitage trend test to evaluate the trend between the scores and MINS, stratified by sex. To quantify the discriminant ability of the scores and the baseline hs-cTnI levels, we used logistic regression to calculate the *C*-statistic with the outcome MINS. We quantified the association between the scores and POD2 hs-cTnI using the Spearman correlation (*r*_s_).

We did several post hoc analyses, which are shown in the supplement. First, we compared categories of hs-cTnI at baseline and POD2 using the Cochran–Armitage test. Next, we used the *R*^2^ calculated from linear regression to quantify the percentage of variability in POD2 hs-cTnI that was explained by one standard deviation (SD) increase in each score. For this regression, the dependent variable of POD2 hs-cTnI was transformed using the natural logarithm because it was right-skewed.

All analyses were two-sided at the alpha level of significance of 0.05 and performed using SAS version 9.4 (SAS Institute Inc., Cary, NC, USA).

## 3. Results

### 3.1. Study Population

Participants (*N* = 808) had a median age of 71 years (IQR 68–75) and were primarily female (63.7%) and Caucasian (91.3%) (Table 1). Most participants (76.2%) underwent total knee arthroplasty and had a history of hypertension (66%). All participants were started on warfarin as thromboprophylaxis, half (48.9%) were taking a statin, and 39% were taking aspirin.

### 3.2. Myocardial Injury after Noncardiac Surgery (MINS)

At baseline, 634 participants (81.9%) had hs-cTnI <5 ng/L, but 16 participants (2%) had hs-cTnI values exceeding the sex-specific 99th percentile thresholds. On POD2, 320 participants (39.6%) had hs-cTnI values greater than or equal to 5 ng/L but lower than or equal to the sex-specific thresholds, and 82 participants (10%) had values exceeding these thresholds (Supplemental Table 1), of whom 78 also exceeded their baseline values, qualifying for MINS. Of the 78 (9.7%) patients with MINS, only three (all female) had clinically identifiable MI during hospitalization. MINS was more common among those with higher triglycerides, elevated baseline hs-cTnI, female sex, and coronary artery disease (Table 1).

### 3.3. Performance of Risk Prediction Tools

In the primary analysis of women, none of the scores significantly predicted MINS, and the *C*-statistics of the scores barely exceeded 0.50 (Table 2). In the primary analysis of men, the ASCVD, ACS-NSQIP, and RCRI scores significantly predicted MINS, with *C*-statistics of 0.66 (95% CI, 0.51–0.82), 0.69 (95% CI, 0.54–0.85), and 0.64 (95% CI, 0.51–0.76). In the categorical analyses (Supplemental Table 2), only RCRI predicted MINS in women (*p*=0.03) and only ASCVD and ACS-NSQIP scores predicted MINS in men (*p*=0.049 and 0.001, respectively). Baseline hs-cTnI was the strongest predictor of MINS in both women and men, with *C*-statistics of 0.63 and 0.72, respectively (Table 2).

All of the risk scores significantly correlated with POD2 hs-cTnI, with correlations (*r*_s_) of 0.24, 0.20, 0.11, 0.11, and 0.08 for the ASCVD, Framingham, ACS-NSQIP, RCRI, and R-RCRI scores (Table 3). However, only the ASCVD score significantly correlated with the rise in hs-cTnI (*r*_s_ = 0.13; *p* < 0.001). The percent of the variability in POD2 hs-cTnI explained by the scores was low, with the highest *R*^2^ (4%) from the ASCVD score (Supplemental Table 3). Combining the scores with the baseline hs-cTnI increased the *R*^2^ to approximately 22% (Supplemental Table 3).

## 4. Discussion

We evaluated how five widely used cardiac risk scores predicted MINS and postoperative hs-cTnI in 808 elderly patients who underwent elective total hip or knee arthroplasty. Similar to previous studies [21, 22], we observed a 10% incidence of MINS, which far exceeded the incidence of identified myocardial infarction (<1%). The risk of MINS was greater among women and in patients with higher triglycerides and a history of CAD. The strong association between CAD and MINS is consistent with previous studies [3, 7, 11, 21, 23, 24]. The modest ability of the risk scores to predict postoperative MINS after arthroplasty is similar to prior evaluations of the ACS-NSQIP [13] and the RCRI in arthroplasty patients [12].

Baseline hs-cTnI was the strongest predictor of MINS and POD2 hs-cTnI. Similarly, in their meta-analysis, Zhao et al. found that baseline preoperative hs-cTnT predicted cardiac complications after noncardiac surgery [25]. Thus, when combined with prior evidence, the current study confirms that preoperative hs-cTn levels predict postoperative myocardial injury. All five cardiac risk scores—ASCVD, FRS, RCRI, R-RCRI, and ACS-NSQIP—correlated with POD 2 hs-cTnI levels. The highest correlation with POD2 hs-cTnI was with the ASCVD score (*r*_s_ = 0.24; *p* < 0.001). Only the ASCVD score was significantly associated with a rise in hs-cTnI (*r*_s_ = 0.13; *p* < 0.001). Thus, the ASCVD score, which was developed to predict 10-year risk in ASCVD events, could potentially be used to predict cardiac complications after arthroplasty but requires further investigation to establish its clinical utility. We are unaware of any prior study using the ASCVD score to predict complications after noncardiac surgery.

This study and prior research studies found that combining a risk score with a biomarker increases predictive accuracy. Here, combining the prediction risk scores with baseline hs-cTnI increased the percent of variability explained (*R*^2^) in POD2 hs-cTnI to approximately 22%. In the vascular events in noncardiac surgery patients cohort evaluation (VISION), adding N-terminal pro-B-type natriuretic peptide (NT-proBNP) to RCRI improved prediction of vascular death and/or MINS from a *C*-statistic of 0.65 to 0.73 [26]. Adding BNP to classic scores improved their discrimination in 242 elderly patients undergoing orthopedic surgery [11]. Likewise, among 227 patients undergoing arthroplasty, RCRI had a *C*-statistic of 0.63, preoperative BNP had a *C*-statistic of 0.77, and the combination improved discrimination (*C*-statistic not reported) [12]. Thus, future research should determine whether ASCVD and other scores should be combined with baseline hs-cTn, NT-proBNP, or BNP to risk stratify patients undergoing noncardiac surgery.

Future research also could determine whether the scores should be combined with functional capacity. One trial found that adding information about functional capacities, such as the ability to climb two flights of stairs, improved discrimination over RCRI alone [27], but subjective functional capacity assessment may not effectively predict cardiac risk [28]. Likewise, comorbidities that are not included in currently used preoperative scores, such as obstructive sleep apnea, might improve their predictive ability [29].

Improvements in risk discrimination are especially needed in women, where all scores were unable to adequately predict MINS. This study used the recommended threshold of hs-cTnI >16 ng/L in women, which improved the sensitivity to detect myocardial injury and infarction in prior research [30]. However, small cTn elevations may be difficult to predict because they are common after stochastic perioperative events, such as hypotension, cardiac arrhythmias, pulmonary embolism, and sepsis [31].

Two prior studies found that the ACS-NSQIP had better discrimination than the RCRI. In a large retrospective study of patients who underwent noncardiac surgery, Glance et al. reported that ACS-NSQIP had superior discrimination for cardiac arrest and/or myocardial infarction (*C*-statistic of 0.81) compared to the RCRI (*C*-statistic 0.68) [32]. However, they used the ACS-NSQIP dataset for their analysis, rather than an independent sample. Cohn and Ros compared several scores in 663 patients who underwent surgery. For the outcome of 30-day major cardiac complications, they reported *C*-statistics of 0.77, 0.55, and 0.56 for the ACS-NSQIP, RCRI, and R-RCRI scores [33]. However, these *C*-statistics were imprecise because only 3 patients had major postoperative cardiac events, and the *C*-statistics were larger when including additional complications (e.g., pulmonary edema and complete heart block) [33]. None of the prior studies examined the ability of the ASCVD score to predict postoperative complications.

Our study has several limitations. First, the homogeneity of the cohort might have contributed to the low predictive ability of the risk scores in this study. Because all participants underwent elective arthroplasty, no participant had high-risk features (i.e., emergency surgery, an American Society of Anesthesiologists (ASA) score of 4 or greater, ventilator dependence, disseminated cancer, or acute renal failure). Second, because the minimum age in our cohort was 65 years, no participant was low risk. Third, electrocardiograms (ECGs) in this study were obtained at the discretion of the attending orthopedists. Had ECGs been done routinely, the incidence of subclinical myocardial infarction and the predictive ability of the scores might have been higher. Additionally, as an ancillary study, the study was limited by the clinical data and the timing of blood specimens collected in the parent clinical trial, GIFT. Thus, we had to use a proxy measure of functional status, which might have handicapped the ACS-NSQIP. We measured hs-cTnI on postoperative day 2; however, daily postoperative measurement of hs-cTnI might have increased the incidence of myocardial infarction and MINS [21, 34, 35] and, possibly, the predictive ability of the scores.

In summary, we evaluated the utility of five widely available scores in the prediction of postoperative hs-cTnI and MINS in elderly patients who underwent elective total hip or knee arthroplasty. Several of the scores modestly predicted MINS in men, but not in women, and all of them correlated with POD2 hs-cTnI. Our results suggest that ASCVD, designed to predict long-term cardiovascular complications in the general population, could be used to predict postoperative troponin levels in men undergoing elective arthroplasty and may be as accurate as the RCRI in this population. Furthermore, investigations in diverse population settings with larger samples are warranted to evaluate its utility in predicting cardiovascular complications after noncardiac surgery.

## Figures and Tables

**Table 1:** Baseline demographic and clinical factors, stratified by myocardial injury on postoperative day (POD) 2.

Parameters	No MINS		MINS		*P* value
	*N* = 730 (90.3%)		*N* = 78 (9.7%)		
	Median (IQR)		Median (IQR)		
Age^*∗*^^‡^ (years)	71 (68, 75)		70 (68, 77)		0.20

BMI (kg/m^2^)	28.2 (24.8, 32.6)		29.0 (24.4, 32.6)		0.67

Creatinine^†^ (mg/dL)	0.8 (0.7, 0.9)		0.8 (0.7, 1.0)		0.62

Cholesterol (total)^*∗*^^‡^ (mg/dL)	157 (139, 179)		153 (135, 172)		0.50

HDL^*∗*^^‡^ (mg/dL)	53 (44, 64)		48 (42, 61)		0.99

Triglyceride (mg/dL)	82 (60, 111)		101 (70, 127)		**0.03**

SBP^*∗*^^‡^	132 (123, 140)		132 (122, 144)		0.30

Baseline hs-cTnI (ng/L)	2.2 (1.5, 3.2)		3.0 (1.7, 5.2)		**0.001**

Sex	N	%	N	%	
Female^*∗*^^‡^	452	61.9	63	80.8	**0.001**

Indication^†^					0.49
Hip arthroplasty	171	23.4	21	26.9	
Knee arthroplasty	559	76.6	57	73.1	

Race^‡^					0.67
White, Caucasian, or Middle Eastern	664	91.0	74	94.9	
African American or Black	41	5.6	3	3.8	
American Indian or Alaska native	1	0.1	0	0.0	
Asian or Indian subcontinent	16	2.2	0	0.0	
Others	8	1.1	1	1.3	

Ethnicity					
Hispanics	22	3.0	2	2.6	1.00

History					
Atrial fibrillation	7	1.0	1	1.3	0.56
Coronary artery disease^*∗*^^†^	59	8.1	13	16.7	**0.01**
Diabetes mellitus^*∗*^^‡^	100	13.8	12	15.4	0.68
Heart failure	11	1.5	1	1.3	1.00
Hypertension	481	65.9	52	66.7	0.89
Liver disease	7	1.0	0	0.0	1.00
Smoking^*∗*^^‡^	26	3.6	1	1.3	0.50
Stroke^†^	9	1.2	1	1.3	1.00
Venous thromboembolism	8	1.1	1	1.3	0.60

Medications					
Antihypertensive^*∗*^^‡^	455	61.9	50	64.1	0.76
Aspirin	278	38.1	37	47.4	0.11
Insulin^†^	10	1.4	3	3.9	0.12
Statin	354	48.5	41	52.6	0.49

BMI, body mass index; HDL, high-density lipoproteins; hs-cTnI, high-sensitivity cardiac troponin I; IQR, interquartile range; MINS, myocardial injury after noncardiac surgery; *N*, number; SBP, systolic blood pressure. Myocardial injury on POD2 was defined by sex-specific 99th percentile. Significant *p* values are highlighted in bold. ^*∗*^Included in Framingham risk score (FRS). ^†^Included in revised cardiac risk index (RCRI). ^‡^Included in atherosclerotic cardiovascular disease risk score (ASCVD).

**Table 2:** *C*-statistics of scores and *p* values for prediction of MINS.

Risk score	Score in women			Score in men		
	*C*-statistic	95% CI	*P* value	*C*-statistic	95% CI	*P* value
ASCVD	0.50	0.42, 0.58	0.98	0.66	0.51, 0.82	0.04
Framingham score	0.52	0.44, 0.59	0.71	0.61	0.46, 0.76	0.14
ACS-NSQIP	0.53	0.45, 0.60	0.55	0.69	0.54, 0.85	0.01
RCRI	0.53	0.49, 0.58	0.13	0.64	0.51, 0.76	0.04
R-RCRI	0.53	0.49, 0.57	0.17	0.61	0.48, 0.74	0.09
Baseline hs-cTnI	0.63	0.55, 0.71	0.001	0.72	0.56, 0.88	0.01

ACS-NSQIP, American College of Surgeon's National Surgical Quality Improvement Program; ASCVD, atherosclerotic cardiovascular disease calculator; RCRI, the revised cardiac risk index; R-RCRI, the reconstructed RCRI.

**Table 3:** Spearman correlations (*r*_s_) of score vs. POD2 hs-cTnI.

Risk score	Correlations vs. POD2 hs-cTnI		Correlations vs. Δ hs-cTnI	
	*r*_s_	*P* value	*r*_s_	*P* value
ASCVD	0.24	<0.001	0.13	<0.001
Framingham score	0.20	<0.001	0.06	0.07
ACS-NSQIP	0.11	<0.001	0.01	0.83
RCRI	0.11	0.006	0.06	0.07
R-RCRI	0.08	0.025	0.05	0.15
Baseline hs-cTnI	0.51	0.001	–0.02	0.54

ACS-NSQIP, American College of Surgeon's National Surgical Quality Improvement Program; ASCVD, atherosclerotic cardiovascular disease calculator; Δ hs-cTnI, change in high-sensitivity cardiac troponin I; POD2, postoperative day 2; RCRI, the revised cardiac risk index; R-RCRI, the reconstructed RCRI.

## Data Availability

No data were used to support this study.

## Supplementary Materials

Supplementary Materials Supplemental Table 1: high-sensitivity cardiac troponin I (hs-cTnI) concentrations by category. POD2, postoperative day 2. ^*∗*^24 participants had insufficient baseline plasma specimens. ^†^Sex-specific 99^th^ percentile cutoff (16 ng/L for women, 34 ng/L for men). Supplemental Table 2: risk scores and association with MINS, stratified by sex. ACS-NSQIP, American College of Surgeon's National Surgical Quality Improvement Program; ASCVD, atherosclerotic cardiovascular disease calculator; MINS, myocardial injury after noncardiac surgery; RCRI, the revised cardiac risk index; R-RCRI, the reconstructed RCRI. *p* value, Cochran–Armitage trend test. Supplemental Table 3: effect of risk score and baseline hs-cTnI on POD2 hs-cTnI, model *R*^2^. ACS-NSQIP, American College of Surgeons' National Surgical Quality Improvement Program; ASCVD, atherosclerotic cardiovascular disease calculator; CAD, coronary artery disease; hs-cTnI, high-sensitivity cardiac troponin I; NA, not applicable; RCRI, the revised cardiac risk index; R-RCRI, the reconstructed RCRI.
