# Supplementary material for: Predicting Postoperative Troponin in Patients Undergoing Elective Hip or Knee Arthroplasty: A Comparison of Five Cardiac Risk Prediction Tools
Source: Cardiol Res Pract. 2022 Oct 14;2022:8244047. doi: 10.1155/2022/8244047 (PMC9586832; doi:10.1155/2022/8244047)
Supplement: Supplementary Materials — Supplemental Table 1: high-sensitivity cardiac troponin I (hs-cTnI) concentrations by category. POD2, postoperative day 2. ∗24 participants had insufficient baseline plasma specimens. †Sex-specific 99th percentile cutoff (16 ng/L for women, 34 ng/L for men). Supplemental Table 2: risk scores and association with MINS, stratified by sex. ACS-NSQIP, American College of Surgeon's National Surgical Quality Improvement Program; ASCVD, atherosclerotic cardiovascular disease calculator; MINS, myocardial injury after noncardiac surgery; RCRI, the revised cardiac risk index; R-RCRI, the reconstructed RCRI. p value, Cochran–Armitage trend test. Supplemental Table 3: effect of risk score and baseline hs-cTnI on POD2 hs-cTnI, model R2. ACS-NSQIP, American College of Surgeons' National Surgical Quality Improvement Program; ASCVD, atherosclerotic cardiovascular disease calculator; CAD, coronary artery disease; hs-cTnI, high-sensitivity cardiac troponin I; NA, not applicable; RCRI, the revised cardiac risk index; R-RCRI, the reconstructed RCRI. [file 8244047.f1.docx]

**Supplemental Table 1: High sensitivity cardiac troponin I (hs-cTnI) concentrations by category.**

| hs-cTnI (ng/L) | Baseline* | POD2 | P Value |
| --- | --- | --- | --- |
|  | **N (%)** | **N (%)** |  |
| < 5 | 634 (81.9) | 406 (50.2) | **<0.0001** |
| 5 ≤ hs-cTnI ≤ 16/34^†^ | 135 (17.2) | 320 (39.6) |  |
| > 16/34 | 15 (1.9) | 82 (10.1) |  |
| Total 784 808 | | | |

POD2, postoperative day 2.

*24 participants had insufficient baseline plasma specimens;

†Sex specific 99^th^ percentile cutoff (16 ng/L for women, 34 ng/L for men).

**Supplemental Table 2: Risk Scores and Association with MINS, Stratified by Sex**

|  | **Women** | |  | **Men** | |  |
| --- | --- | --- | --- | --- | --- | --- |
| **Score** | **No MINS, N** | **MINS, N** | **P Value** | **No MINS, N** | **MINS, N** | **P Value** |
| **ASCVD** |  |  | 0.47 |  |  | **0.049** |
| 0 - 9.9% | 139 | 19 |  | 11 | 0 |  |
| 10 - 19.9% | 171 | 22 |  | 93 | 4 |  |
| 20 - 29.9% | 71 | 12 |  | 83 | 4 |  |
| 30 - 39.9% | 37 | 3 |  | 48 | 1 |  |
| 40 - 49.9% | 20 | 2 |  | 26 | 3 |  |
| 50 - 100% | 14 | 5 |  | 17 | 3 |  |
| **Framingham Risk** |  |  | 0.74 |  |  | 0.26 |
| 0-9.9% | 207 | 28 |  | 7 | 0 |  |
| 10-19.9% | 188 | 28 |  | 78 | 3 |  |
| 20-29.9% | 39 | 6 |  | 88 | 5 |  |
| 30-39.9% | 13 | 1 |  | 53 | 2 |  |
| 40-49.9% | 4 | 0 |  | 28 | 3 |  |
| 50-100% | 1 | 0 |  | 24 | 2 |  |
| **ACS-NSQIP** |  |  | 0.48 |  |  | **0.001** |
| 0% | 169 | 22 |  | 87 | 2 |  |
| 0.1-0.2% | 187 | 25 |  | 95 | 4 |  |
| 0.3-0.4% | 79 | 13 |  | 51 | 0 |  |
| 0.5-0.6% | 14 | 2 |  | 30 | 5 |  |
| 0.7-1.4% | 3 | 1 |  | 15 | 4 |  |
| **RCRI** |  |  | **0.03** |  |  | 0.13 |
| 0 | 424 | 55 |  | 229 | 8 |  |
| 1 | 24 | 6 |  | 37 | 7 |  |
| 2 | 3 | 1 |  | 10 | 0 |  |
| 3 | 1 | 1 |  | 2 | 0 |  |
| **R-RCRI** |  |  | 0.07 |  |  | 0.17 |
| 0 | 427 | 56 |  | 232 | 9 |  |
| 1 | 22 | 5 |  | 35 | 6 |  |
| 2 | 2 | 2 |  | 11 | 0 |  |
| 3 | 1 | 0 |  | 0 | 0 |  |

ACS-NSQIP American College of Surgeon’s National Surgical Quality Improvement Program; ASCVD, atherosclerotic cardiovascular disease calculator; MINS, myocardial injury after noncardiac surgery; RCRI, the revised cardiac risk index; R-RCRI, the reconstructed RCRI. P-value, Cochran–Armitage trend test.

| **Supplemental Table 3: Effect of Risk Score and baseline hs-cTnI on POD2 hs-cTnI, Model R^2^** | | | | | | |
| --- | --- | --- | --- | --- | --- | --- |
| **Variables in Regression Model** | **ASCVD** | **Framingham Score** | **NSQIP** | **RCRI** | **R-RCRI** | **Baseline hs-cTnI** |
| Score | 4.0% | 1.8% | 2.6% | 1.7% | 1.2% | 20.6% |
| Score, aspirin, statin, CAD | 5.6% | 3.6% | 3.6% | 2.6% | 2.5% | 21.9% |
| Score, aspirin, statin, CAD, baseline hs-cTnI | 22.4% | 21.9% | 22.0% | 21.9% | 21.9% | NA |

ACS-NSQIP American College of Surgeon’s National Surgical Quality Improvement Program; ASCVD, atherosclerotic cardiovascular disease calculator; CAD, coronary artery disease; hs-cTnI, high sensitivity troponin I; NA, not applicable. RCRI, the revised cardiac risk index; R-RCRI, the reconstructed RCRI.
